# Supplementary material for: Efficacy and Safety of Acupuncture and Related Techniques in the Management of Oncological Children and Adolescent Patients: A Systematic Review
Source: Cancers (Basel). 2024 Sep 19;16(18):3197. doi: 10.3390/cancers16183197 (PMC11429796; doi:10.3390/cancers16183197)
Supplement: Supplementary file 1 [file cancers-16-03197-s001.zip › cancers-3182883-supplementary.pdf]

Supplementary materials

Supplement S1: Search strategies

PUBMED

1. "acupuncture therapy"[MeSH Terms]
2. "acup*"[Title/Abstract]
3. "moxibustion"[MeSH Terms]
4. "Moxibustion"[Title/Abstract]
5. "Moxa"[Title/Abstract]
6. "electroacup*"[Title/Abstract]
7. "acupuncture, ear"[MeSH Terms]
8. "press tack"[Title/Abstract]
9. (1-8) OR
10. "pediatrics"[MeSH Terms]
11. "child*"[Title/Abstract]
12. "pediatr*"[Title/Abstract]
13. "Adolescent"[Title/Abstract]
14. (10-13) OR
15. "neoplasms"[MeSH Terms]
16. "Neoplasm"[Title/Abstract]
17. "Cancer"[Title/Abstract]
18. "Oncology"[Title/Abstract]
19. "leukemi*"[Title/Abstract]
20. "Tumor"[Title/Abstract]
21. "Sarcoma"[Title/Abstract]
22. "chemotherap*"[Title/Abstract]
23. "radiotherap*"[Title/Abstract]
24. "immunotherap*"[Title/Abstract]
25. (15-24) OR
26. "randomized controlled trial"[Publication Type]
27. "clinical trial"[Publication Type]
28. "Randomized"[Title/Abstract]
29. "Randomised"[Title/Abstract]
30. "Randomly"[Title/Abstract]
31. "Trial"[Title/Abstract]
32. "Control"[Title/Abstract]
33. "Controlled"[Title/Abstract]
34. (26-33) OR
35. 9 AND 14 AND 25 AND 34

COCHRANE

#1 MeSH descriptor: [Acupuncture Therapy] explode all trees
#2 (acup*):ti,ab,kw
#3 MeSH descriptor: [Moxibustion] explode all trees
#4 (moxibustion):ti,ab,kw
#5 (moxa):ti,ab,kw
#6 (electroacup*):ti,ab,kw
#7 MeSH descriptor: [Acupuncture, Ear] explode all trees
#8 (press tack):ti,ab,kw
#9 #1 OR #2 OR #3 OR #4 OR #5 OR #6 OR #7 OR #8
#10 MeSH descriptor: [Pediatrics] explode all trees
#11 (child*):ti,ab,kw
#12 (pediatr*):ti,ab,kw
#13 (adolescent*):ti,ab,kw
#14 #10 OR #11 OR #12 OR #13
#15 MeSH descriptor: [Neoplasms] explode all trees
#16 (neoplasm):ti,ab,kw
#17 (cancer):ti,ab,kw
#18 (oncology):ti,ab,kw
#19 (leukemi*):ti,ab,kw
#20 (tumor):ti,ab,kw
#21 (sarcoma):ti,ab,kw
#22 (chemotherap*):ti,ab,kw
#23 (radiotherap*):ti,ab,kw
#24 (immunotherap*):ti,ab,kw
#25 #15 OR #16 OR #17 OR #18 OR #19 OR #20 OR #21 OR #22 OR #23 OR #24
#26 (randomized):ti,ab,kw
#27 (randomised):ti,ab,kw
#28 (randomly):ti,ab,kw
#29 (trial):ti,ab,kw
#30 (control):ti,ab,kw
#31 (controlled):ti,ab,kw
#32 #26 OR #27 OR #28 OR #29 OR #30 OR #31
#33 #9 AND #14 AND #25 AND #32

PEDRO

Abstract & Title: children

Therapy: acupuncture

Subdiscipline: oncology

Method: clinical trial

CINHAL COMPLETE

#	Search
S5	S1 AND S2 AND S3 AND S4
S4	PT randomized controlled trials OR AB randomized OR AB randomised OR AB randomly OR AB trial OR AB control OR AB controlled OR PT clinical trial
S3	MH neoplasms OR AB (neoplasms or oncology or cancer) OR AB leukemia OR AB tumor OR AB sarcoma OR AB (chemotherapy or chemo or cancer treatment) OR AB (radiotherapy or radiation therapy) OR immunotherapy
S2	MH pediatrics OR AB (pediatrics or children) OR AB adolescents
S1	MH acupuncture therapy OR AB acupuncture OR AB moxibustion OR AB moxa OR AB (electroacupuncture or electro-acupuncture) OR MH acupuncture, ear OR press tack

EMBASE

1	'acupuncture'/exp OR 'moxibustion'/exp OR 'electroacupuncture'/exp OR 'auricular acupuncture'/exp OR (acupuncture:ti,ab,kw OR 'press tack':ti,ab,kw) AND moxa:ti,ab,kw
2	'pediatrics'/exp OR 'child'/exp OR 'adolescent'/exp OR child:ti,ab,kw OR children:ti,ab,kw OR pediatric:ti,ab,kw OR adolescent:ti,ab,kw
3	'neoplasm'/exp OR 'cancer therapy'/exp OR 'oncology'/exp OR neoplasm:ti,ab,kw OR cancer:ti,ab,kw OR oncology:ti,ab,kw OR leukemia:ti,ab,kw OR 'tumor'/exp OR tumor OR 'sarcoma'/exp OR sarcoma OR 'chemotherapy'/exp OR chemotherapy OR 'radiotherapy'/exp OR radiotherapy OR 'immunotherapy'/exp OR immunotherapy OR 'lymphoma'/exp OR lymphoma
4	1 AND 2 AND 3
5	'clinical study'/exp OR 'randomized controlled trial'/exp OR 'controlled study'/exp OR 'randomized controlled trial':ti,ab,kw OR 'clinical study':ti,ab,kw OR 'controlled study':ti,ab,kw OR randomized:ti,ab,kw OR randomised OR randomly OR 'trial'/exp OR trial OR 'control'/exp OR control OR controlled OR random
6	4 AND 5

CNKI

1	TI/KY/AB= 针灸 + 针刺 + 灸 + 揿针 + 电针 + 耳针 + 耳穴
2	TI/KY/AB= 婴儿 + 幼儿 + 婴幼儿 + 小儿 + 孩子 + 小孩 + 孩童 + 儿童 + 儿科 + 青少年 + 少年 + 未成年
3	TI/KY/AB= 癌 + 瘤 + 肉瘤 + 白血病 + 化疗 + 靶向 + 放疗 + 免疫疗法
4	TI/KY/AB= 临床研究 + 试验 + 疗效观察 + 疗效评价 + 临床观察
5	TI/KY/AB= 随机+对照
6	1 AND 2 AND 3 AND 4 AND 5

WANFANG DATA

1	主题: (针灸 + 针刺 + 灸 + 揿针 + 电针 + 耳针 + 耳穴)
2	主题: (婴儿 + 幼儿 + 婴幼儿 + 小儿 + 孩子 + 小孩 + 孩童 + 儿童 + 儿科 + 青少年 + 少年 + 未成年)
3	主题: (癌 + 瘤 + 肉瘤 + 白血病 + 化疗 + 靶向 + 放疗 + 免疫疗法)
4	主题: (临床研究 + 试验 + 疗效观察 + 疗效评价 + 临床观察)
5	主题: (随机+对照)
6	1 AND 2 AND 3 AND 4 AND 5

VIP

1	题名或关键词或摘要= (针灸 + 针刺 + 灸 + 揿针 + 电针 + 耳针 + 耳穴)
2	题名或关键词或摘要= (婴儿 + 幼儿 + 婴幼儿 + 小儿 + 孩子 + 小孩 + 孩童 + 儿童 + 儿科 + 青少年 + 少年 + 未成年)
3	题名或关键词或摘要= (癌 + 瘤 + 肉瘤 + 白血病 + 化疗 + 靶向 + 放疗 + 免疫疗法)
4	题名或关键词或摘要= (临床研究 + 试验 + 疗效观察 + 疗效评价 + 临床观察)
5	题名或关键词或摘要= (随机+对照)
6	1 AND 2 AND 3 AND 4 AND 5

SINOMED

1	题名或关键词或摘要= (针灸 + 针刺 + 灸 + 揿针 + 电针 + 耳针 + 耳穴)
2	题名或关键词或摘要= (婴儿 + 幼儿 + 婴幼儿 + 小儿 + 孩子 + 小孩 + 孩童 + 儿童 + 儿科 + 青少年 + 少年 + 未成年)
3	题名或关键词或摘要= (癌 + 瘤 + 肉瘤 + 白血病 + 化疗 + 靶向 + 放疗 + 免疫疗法)
4	题名或关键词或摘要= (临床研究 + 试验 + 疗效观察 + 疗效评价 + 临床观察)
5	题名或关键词或摘要= (随机+对照)
6	1 AND 2 AND 3 AND 4 AND 5

Supplement S2: List of excluded trials with reasons

Wrong study design (n=2):

Hu H, Shear D, Thakkar R, Thompson-Lastad A, Pinderhughes H, Hecht F, et al. Acupressure and Therapeutic Touch in Childhood Cancer to Promote Subjective and Intersubjective Experiences of Well-being During Curative Treatment. Global advances in health and medicine [Internet]. 2019;8. Available at: https://www.cochranelibrary.com/central/doi/10.1002/central/CN-01997083/full

袁嘉.浅述穴位疗法缓解急性白血病化疗患儿胃肠道反应的临床效果[J].中西医结合心血管病电子杂志,2017,5(02):35-36.DOI:10.16282/j.cnki.cn11-9336/r.2017.02.025.

Wrong intervention (n=4):

Mehling W, Lown E, Dvorak C, Cowan M, Horn B, Dunn E, et al. Hematopoietic cell transplant and use of massage for improved symptom management: results from a pilot randomized control trial. Evidence-based complementary and alternative medicine [Internet]. 2012; Disponible en: https://www.cochranelibrary.com/central/doi/10.1002/central/CN-00897044/full

Essawy MA, Abohadida RM, Abd-Elkader WM, Fathy HM, Hassab HM. Comparing the effect of acupressure and ginger on chemotherapy gastrointestinal side-effects in children with leukemia. Complement Ther Med [Internet]. 2021;60. Disponible en: https://www.embase.com/search/results?subaction=viewrecord&id=L2013048622&from=export U2 - L2013048622

王燕,张红.中医护理在白血病患儿化疗中的应用效果[J].当代护士(中旬刊),2015(01):48-49.

辛育龄, **刘德若**, 孟新, 李福田, 张伟, 赵洪昌, et al. 电针治疗儿童海绵状血管瘤的临床观察. 中华小儿外科杂志. 2002;23(5):465-6.

Wrong population (n=16):

An L, He Y, Ren X, Li S, Han R, Wang B. Effect of electroacupuncture on Sevoflurane anesthesia in patients undergoing resection of supratentorial tumor. Zhongguo zhenjiu [Chinese acupuncture & moxibustion]. 2010;30(8):669-673.

An L, Li J, Ren X, Liu Y, Wang B. Effects of electroacupuncture of different acupoint groups on sevoflurane anesthesia in patients undergoing resection of supratentorial tumors. Zhen CI yan jiu = acupuncture research. 2010;35(5):368-374.

Avc H, Ovayolu N, Ovayolu Ö. Effect of Acupressure on Nausea-Vomiting in Patients With Acute Myeloblastic Leukemia. Holistic nursing practice. 2016;30(5):257-262.

Chen J, Zhang Y, Li X, Wan Y, Ji X, Wang W, et al. Efficacy of transcutaneous electrical acupoint stimulation combined with general anesthesia for sedation and postoperative analgesia in minimally invasive lung cancer surgery: a randomized, double-blind, placebo-controlled trial. Thoracic cancer. 2020;11(4):928-934.

Chen H, Liu T, Kuai L, Zhu J, Wu C, Liu L. Electroacupuncture treatment for pancreatic cancer pain: a randomized controlled trial. Pancreatology. 2013;13(6):594-597.
Feng Y, Wang X, Li S, Zhang Y, Wang H, Li M, et al. Clinical research of acupuncture on malignant tumor patients for improving depression and sleep quality. Journal of traditional chinese medicine = chung i tsa chih ying wen pan. 2011;31(3):199-202.

Chen X, Wang B, Li J, An L. Effect of transcutaneous acupoint electric stimulation on perioperative intravenous anesthesia in patients of transsphenoidal pituitary tumorresection. Zhongguo zhen jiu [Chinese acupuncture & moxibustion]. 2013;33(8):732-736.

Fang Y, Deshan L, Shuli W, Lan X. Effects of electro-acupuncture on T cell subpopulations, NK activity, humoral immunity and leukocyte count in patients undergoing chemotherapy. J Trad Chin Med. 2007;27(1):19–21.

Fu J, Meng Z, Chen Z, Peng H, Liu L. Clinical observation on electric stimulation of Yongquan (KI 1) for prevention of nausea and vomiting induced by Cisplatin. Zhongguo zhen jiu [Chinese acupuncture & moxibustion]. 2006;26(4):250-252.

Melchart D, Ihbe-Heffinger A, Leps B, von Schilling C, Linde K. Acupuncture and acupressure for the prevention of chemotherapy-induced nausea--a randomised cross-over pilot study. Supportive care in cancer. 2006;14(8):878-882.

Mohammed HG, Al-Sharkawi SS, Mohammed Adly R. The Effect of an Acupressure Training Program for Pediatric Nurses Caring for Children Undergoing Chemotherapy. Plast Aesthet Nurs (Phila). 2022 Oct-Dec 01;42(4):197-205. doi: 10.1097/PSN.0000000000000463. PMID: 36469390.

Molassiotis A, Russell W, Hughes J, Breckons M, Lloyd-Williams M, Richardson J, et al. The effectiveness of acupressure for the control and management of chemotherapy-related acute and delayed nausea: a randomized controlled trial. Journal of pain and symptom management. 2014;47(1):12-25.

Molassiotis A, Russell W, Hughes J, Breckons M, Lloyd-Williams M, Richardson J, et al. The effectiveness and cost-effectiveness of acupressure for the control and management of chemotherapy-related acute and delayed nausea: assessment of Nausea in Chemotherapy Research (ANCHoR), a randomised controlled trial. Health technology assessment (Winchester, England). 2013;17(26):1-114

Sima L, Wang X. Therapeutic effect of acupuncture on cisplatin-induced nausea and vomiting. Zhongguo zhen jiu [Chinese acupuncture & moxibustion]. 2009;29(1):3-6.

Yang Y, Zhang Y, Jing N, Lu Y, Xiao H, Xu G, et al. Electroacupuncture at Zusanli (ST 36) for treatment of nausea and vomiting caused by the chemotherapy of the malignant tumor: a multicentral randomized controlled trial. Zhongguozhen jiu [Chinese acupuncture & moxibustion]. 2009;29(12):955-958.

Zhang S, Wu T, Zhang H, Yang Y, Jiang H, Cao S, et al. Effect of electroacupuncture on chemotherapy-induced peripheral neuropathy in patients with malignant tumor: a single-blinded, randomized controlled trial. Journal of traditional chinese medicine = chung i tsa chih ying wen pan. 2017;37(2):179-184.

Zhang X, Fan Y. Effects of electroacupuncture on chemotherapy-induced nausea and vomiting and its mechanism. Zhongguo zhen jiu [Chinese acupuncture & moxibustion]. 2014;34(11):1061-1064.

Wrong publication type (congress presentation or poster, protocol, …) (n=7):

Beate W, Christina S, Nils L, Yanjun L i u, Angelika E, Bernhard K, et al. A randomised placebo-controlled pilot study of pericardium 6 acupressure and acupuncture as additive antiemetic therapy during chemotherapy in children and adolescents. Pediatric blood & cancer. 2008;50(5):171.

Kim B, Kim H, Oh J, Hwang H, Jang H, Choi H, et al. The effects of acupuncture on the insomnia, anxiety and depression of patients with hepatocellular carcinoma. Hepatology international. 2014;8(1):S272.

Lown A, Dvorak C, Acree M, Dunn E, Abrahms D, Horn B, et al. Massage provides relief for symptoms in pediatric hematopoietic transplant patients. Psycho-oncology. 2011;20(1):70-71.

Lown E, Banerjee A, Dvorak C, Hartogensis W, Melton A, Mangurian C, et al. Acupressure to reduce symptoms of depression and anxiety in children in treatment for a childhood cancer and recipients of a hematopoietic stem cell transplant. Psycho-oncology. 2020;29:104-.

Lown A, Banerjee A, Dvorak C, Hartogensis W, Vittinghoff E, Melton A, et al. Acupressure to reduce nausea and vomiting in youth in treatment for cancer or receiving hematopoietic stem cell transplant: a randomized trialdespite improved pharmacologic management of nausea and vomiting, these symptoms remain a problem for children, adolescents, and young adults in treatment for cancer or receiving a hematopoietic stem cell transplant. This study tests whether acupressure reduces symptoms of nausea and vomiting. Global advances in health and medicine. 2020;9:5-6.

Lo L. Effects of P6 acupressure on acute and delayed nausea and vomiting in children receiving cancer chemotherapy. [Internet]. Effects of P6 Acupressure on Acute & Delayed Nausea & Vomiting in Children Receiving Cancer Chemotherapy. Case Western Reserve University

(Health Sciences); 1998. Available at: https://search.ebscohost.com/login.aspx?direct=true&AuthType=ip,sso&db=ccm&AN=109878332&lang=es&site=ehost-live&custid=ns130442

NCT01346267. Acupressure in Controlling Nausea in Young Patients Receiving Highly Emetogenic Chemotherapy. https://clinicaltrials.gov/show/NCT01346267 [Internet]. 2011; Available at: https://www.cochranelibrary.com/central/doi/10.1002/central/CN-01486282/full

Wrong study outcomes (n=1)

Zhou Y, Li Z, Wang L, Song J, Liu J, Zhou T. Observation of the clinical effects of acupuncture intervention in primary liver cancer in children and the effects on immune function and metabolic function of intestinal flora. Chin J Integr Trad West Med Dig. 2022;30(12):854–8.

Duplicate (n=2)

Bastani F, Khosravi M, Borimnejad L, Arbabi N. The effect of acupressure on cancer-related fatigue among school-aged children with acute lymphoblastic leukemia. Iran J Nurs Midwifery Res. 2015;20(5):545-51. (Same data as Ghezelbash 2017)

Reindl T, Geilen W, Hartmann R, Wiebelitz K, Kan G, Wilhelm I, et al. Acupuncture against chemotherapy-induced nausea and vomiting in pediatric oncology. Interim results of a multicenter crossover study. Supportive care in cancer. 2006;14(2):172-176. (Preliminary results from Gottschling S et al.)

Supplement S3: Expended Risk of Bias of individual studies

	Selection bias				Performance bias				Detection bias		Attrition bias		Reporting bias	
**Author**<br><br>**Year**	**Random sequence generation**	**Rationale**	**Allocation concealment**	**Rationale**	**Blinding of participants**	**Rationale**	**Blinding of personnel**	**Rationale**	**Blinding (outcome assessment)**	**Rationale**	**Incomplete outcome data**	**Rationale**	**Selective reporting**	**Rationale**
**Altuntas 2022**	HIGH	Real and sham interventions are not randomized	UNCLEAR	Not reported	LOW	Sham interventions are used	HIGH	Not possible	LOW	Self-reported by blinded participants	LOW	Only 1 loss for each group	LOW	Protocol available NCT04651608
**Bintoro 2022**	LOW	based on a computer-based random table	UNCLEAR	Not reported	LOW	Sham intervention used	HIGH	Not possible	LOW	Blinded participants and data collectors	UNCLEAR	Not reported	UNCLEAR	Protocol not available
**Bai**<br><br>**2024**	LOW	Random table	UNCLEAR	Not reported	HIGH	Not blinded	HIGH	Not blinded	UNCLEAR	Not reported	LOW	No looses	UNCLEAR	Protocol not available
**Dupuis**<br><br>**2018**	LOW	Computer generated	UNCLEAR	Not reported	LOW	Sham bands are used	HIGH	Not possible	LOW	Self-reported by blinded participants	LOW	Intention to treat analysis	LOW	Protocol available NCT01346267
**Ghezelbasch**<br><br>**2017**	LOW	Random table	UNCLEAR	Not reported	LOW	Different acupoints	HIGH	Not blinded	UNCLEAR	Not reported	LOW	No looses	UNCLEAR	Protocol not available
**Gottschling**<br><br>**2008**	LOW	Computerized table of random numbers.	LOW	Centrally performed randomization	HIGH	Not blinded	HIGH	Not blinded	UNCLEAR	Not reported	LOW	No looses	UNCLEAR	Protocol not available

**Jones 2008**	UNCLEAR	Not reported	UNCLEAR	Not reported	LOW	Sham bands	HIGH	Not reported	UNCLEAR	Not reported	HIGH	21/18 (14%) completed	UNCLEAR	Protocol not available
**Liu 2017**	UNCLEAR	Randomized but not reported	UNCLEAR	Not reported	HIGH	Not blinded	HIGH	Not blinded	UNCLEAR	Not reported	LOW	No looses	UNCLEAR	Protocol not available
**Varejão 2019**	UNCLEAR	Participants chose between two envelopes containing either "A" or "B,"	UNCLEAR	Not reported	LOW	Sham laser	HIGH	Not possible	LOW	Self-reported	UNCLEAR	Not reported	UNCLEAR	Protocol not available
**Xie 2016**	LOW	Random table	UNCLEAR	Not reported	HIGH	Not blinded	HIGH	Not blinded	UNCLEAR	Not reported	LOW	102/104 (98.1%)	UNCLEAR	Protocol not available
**Yeh 2012**	LOW	Computer-generated	UNCLEAR	Not reported	LOW	Sham treatment	HIGH	Not blinded	LOW	Self-reported	HIGH	17/10 (41%) completed.	UNCLEAR	Protocol not available

Supplement S4: Sensitivity analysis

Article excluded	I^2	Std. MD [95%CI]
**Nausea and vomiting**		
Altuntas 2022 (Manual)	0%	-0.58 [-0.86, -0.29]
Altuntas 2022 (Wristband)	0%	-0.59 [-0.87, -0.31]
Ghezelbasch 2017	0%	-0.46 [-0.82, -0.10]
Jones 2008	0%	-0.61 [-0.89, -0.34]
**Vomiting episodes**		
Altuntas 2022 (Manual)	0%	-0.18 [-0.46, 0.10]
Altuntas 2022 (Wristband)	0%	-0.16 [-0.41, 0.08]
Jones 2008	0%	-0.15 [-0.35, 0.04]
